# Efficient Cross-Modality Insulator Augmentation for Multi-Domain Insulator Defect Detection in UAV Images

**DOI:** 10.3390/s24020428

**Published:** 2024-01-10

**Authors:** Yue Liu, Xinbo Huang

**Affiliations:** 1 School of Electrical Engineering, Xi’an University of Technology, Xi’an 710054, China; yliu@stu.xaut.edu.cn; 2 School of Electronics and Information, Xi’an Polytechnic University, Xi’an 710048, China

**Keywords:** transmission line inspection, insulator defect detection, unmanned aerial vehicle (UAV), deep learning

## Abstract

Regular inspection of the insulator operating status is essential to ensure the safe and stable operation of the power system. Unmanned aerial vehicle (UAV) inspection has played an important role in transmission line inspection, replacing former manual inspection. With the development of deep learning technologies, deep learning-based insulator defect detection methods have drawn more and more attention and gained great improvement. However, former insulator defect detection methods mostly focus on designing complex refined network architecture, which will increase inference complexity in real applications. In this paper, we propose a novel efficient cross-modality insulator augmentation algorithm for multi-domain insulator defect detection to mimic real complex scenarios. It also alleviates the overfitting problem without adding the inference resources. The high-resolution insulator cross-modality translation (HICT) module is designed to generate multi-modality insulator images with rich texture information to eliminate the adverse effects of existing modality discrepancy. We propose the multi-domain insulator multi-scale spatial augmentation (MMA) module to simultaneously augment multi-domain insulator images with different spatial scales and leverage these fused images and location information to help the target model locate defects with various scales more accurately. Experimental results prove that the proposed cross-modality insulator augmentation algorithm can achieve superior performance in public UPID and SFID insulator defect datasets. Moreover, the proposed algorithm also gives a new perspective for improving insulator defect detection precision without adding inference resources, which is of great significance for advancing the detection of transmission lines.

## 1. Introduction

The safe and stable operation of the power grid has a significant impact on social production and daily life, and ensuring the stable operation of the power system is an important prerequisite for power transmission. The three major channels of west-to-east power transmission implemented by the state grid connect the electricity of five provinces tightly through high-voltage transmission lines. The abundant energy in western provinces is converted into electrical energy resources and then transmitted to the power-scarce eastern coastal areas through high-voltage transmission lines, improving the power grid’s disaster resistance and reduction capabilities. Insulators are an indispensable component in transmission lines that support the line and provide insulation, among other functions. The most commonly used insulators for transmission lines on the power grid are glass disk-type suspension insulators and composite insulators (also known as synthetic insulators). Insulators are subjected to long-term load operation in transmission lines and are greatly affected by natural factors and climate. Insulating materials are highly susceptible to environmental and material aging, resulting in varying degrees of damage. When insulators have self-explosion defects, if they are not handled in a timely manner, they may lead to insulator fractures, impacting insulators such as towers, affecting the reliability of transmission lines, and even causing widespread power outages in the power grid, resulting in significant economic losses [1]. Therefore, the timely detection and accurate positioning of the status of insulator defects [2,3,4,5] are important means to ensure the normal operation of transmission lines.

The traditional manual inspection model has low work efficiency, and the surrounding area of high-voltage transmission lines has harsh environmental conditions and inconvenient transportation, which requires a large risk and cannot fully meet the needs of China’s intelligent power grid construction. With the rapid development of smart grids, as well as the widespread use of computers and intelligent devices, the inspection model using power vision technology for visual processing and analysis of transmission lines has gradually become mainstream [6,7]. Therefore, as a low-cost, short-cycle, and highly maneuverable inspection method, drones are receiving increasing attention in power transmission line inspection [8,9,10]. By processing the images of transmission lines obtained through unmanned aerial vehicle (UAV) inspection, it is possible to accurately detect hidden dangers in transmission lines, reduce the incidence of accidents, and improve the reliability of the power grid operating environment [11,12,13]. Processing and analyzing images captured by UAVs can effectively detect the positions of electrical components and provide technical support for autonomous inspections of transmission line components using UAVs, the autonomous navigation of UAVs, the automatic focusing of cameras, and defect identification. However, UAV inspections inevitably generate a massive amount of inspection images. To further improve the automation level of transmission line inspection, it is necessary to study the recognition and defect detection algorithms for electrical equipment in aerial photos of the UAVs. In [14], a summary of popular deep learning-based insulator defect detection methods is provided. Many effective detection methods are tested on self-built datasets, making replication work complex and limiting the applicability of models, resulting in poor generalization ability. Insulator defect detection models trained on public datasets perform well only on the dataset mentioned in the literature. These datasets have a single-image scale. When the input image size significantly differs from the training image size, the model no longer has a high recognition rate, and its robustness is poor. The datasets used for training only consider good detection results under clear weather conditions and ignore the impact of weather changes on the detection model accuracy in real-world scenarios.

In this paper, we summarize the current status of insulator defect detection tasks based on unmanned aerial vehicles (UAVs) inspection in recent years and propose that there are problems of insufficient detection capability of multi-domain insulators based on visual inspection and missed detection in multi-scale long-range vision. Therefore, we propose a novel efficient cross-modality insulator augmentation algorithm for multi-domain insulator defect detection, which simulates real complex scenarios to compensate for the shortcomings of existing datasets and insulator defect recognition. The algorithm considers multiple perspectives across real-world scenarios in multiple domains, improving the model’s robustness and generalization ability without increasing inference resources.

The main contributions of our paper can be summarized as follows:

1. We explore a novel cross-modality insulator augmentation for a multi-domain insulator defect detection task, which gives a new perspective to improve insulator defect detection precision even in diverse weather conditions.
2. The high-resolution insulator cross-modality translation (HICT) module is designed to generate multiple modality insulator images with diverse weather conditions (e.g., snowy, foggy weather), which can retain rich texture information with high resolution to increase data diversity to mimic real scenarios.
3. The multi-domain insulator multi-scale spatial augmentation (MMA) module is proposed to augment hybrid domain insulator images by fusing both images and bounding boxes in different spatial scales. It can help the detection model locate multi-scale defects more accurately.
4. Experimental results on several public insulator defect datasets (UPID and SFID dataset) illustrate the superior performance of the proposed method compared with comparison methods.

The remainder of this article is organized as follows. Section 2 provides a brief overview of the current state of insulator defect detection and system frameworks. In Section 3, we describe in detail the high-resolution insulator cross-modality translation (HICT) model and multi-domain insulator multi-scale spatial augmentation (MMA) model. Section 4 presents experimental results and analysis. Conclusions are drawn in Section 5.

## 2. Related Work

In the real world, insulators operate outdoors for long periods of time, and weather changes have a significant impact on the operation of transmission line insulators. Therefore, it is very important to conduct drone inspections of insulators promptly [6]. However, due to the confidentiality of power data, there are issues such as the small quantity and imbalance of insulator defect data in the insulator defect detection model, which cannot produce satisfactory results. Data augmentation, as a key technology in image recognition deep learning tasks, can effectively alleviate the data scarcity scenario in insulator defect detection tasks. Liu et al. [15] used noise addition and image rotation to change the style of the original image. Wei et al. [16] extended the images of insulator defects through flipping, translation, and rotation. Li et al. [17] used methods such as rotation, mirroring flipping, contrast, brightness adjustment, and noise pixel generation to address problems of insufficient data quantity and sample imbalance. Liu et al. [18] employed techniques such as cutout, flipping, and color jittering to prevent overfitting. Li et al. [19] used methods such as mirroring flipping, rotation, affine transformation, Gaussian white noise, brightness, and color conversion to address data imbalance issues. Wang et al. [9] proposed an insulator abnormal state detection method for small data samples. Zhang et al. [20], Xin et al. [21] and Liu et al. [22] considered the problem of insulator detection in multiple domains, but they only considered insulator detection in foggy weather and did not deeply investigate insulator detection in other weather conditions. Zhang et al. [20] proposed a fog and clear weather insulator detection model based on a modified Yolov5 network. Liu et al. [22] generated a simulated insulation dataset for foggy weather using the dark channel prior algorithm and improved target detection accuracy in foggy weather environments based on the CenterNet network.

However, in real-world scenarios, natural data exist under various conditions that cannot be explained by these simple methods, such as the season in which the photos were taken, which greatly affects the important features displayed in the photos. Detection models may mistakenly label the target or fail to recognize the target. Therefore, we cannot ignore the impact of season on the model. Currently, the existing datasets have limited reference values in this regard, and multi-domain insulator defect detection [23,24,25] remains an effective topic. Enhancing the insulator data in real-world scenarios remains a problem that needs to be addressed and explored.

To address this problem, we propose a novel efficient cross-modality insulator augmentation algorithm for multi-domain insulator defect detection to mimic real complex scenarios. The high-resolution insulator cross-modality translation (HICT) module is designed to generate multi-modality insulator images with rich texture information to eliminate adverse effects of existing modality discrepancy, and the multi-domain insulator multi-scale spatial augmentation (MMA) module is designed to simultaneously augment multi-domain insulator images with different spatial scales and leverage these fused images and location information to help the target model locate defects with various scales more accurately. This algorithm improves the model’s robustness and generalization ability in real-world scenarios without increasing inference resources, which can inspire more researchers to focus on this field.

## 3. The Proposed Method

**Figure 1:**
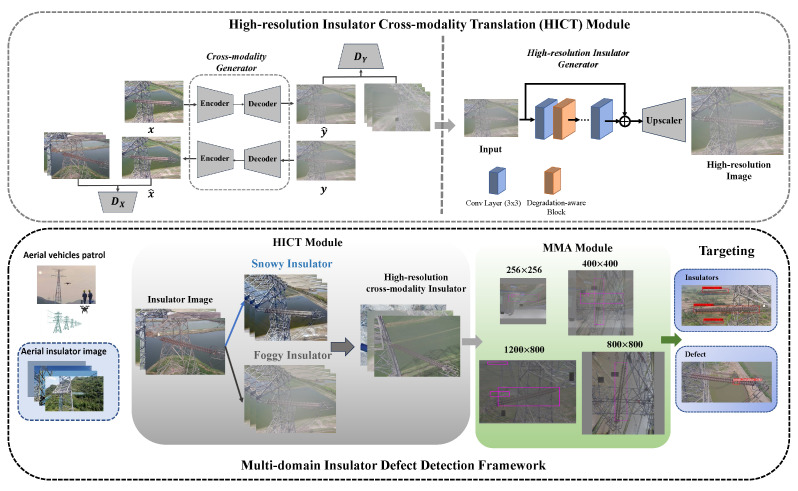
The framework of multi-domain insulator defect detection. The insulator images collected from transmission line UAV inspection are enhanced by the HICT module and MMA module and then input into the detection model for training. The resulting model has the ability for multi-domain insulator defect detection to mimic real complex scenarios.

The confidentiality of power systems makes the insulator dataset have difficulties such as small data volume, imbalanced data samples, and difficulty in acquisition. It is not practical to collect insulator images under different weather conditions in the same scenario. However, deep learning-based detection networks require a large amount of training resources. Therefore, data augmentation methods are key technologies for solving various challenging deep-learning tasks. In this paper, we propose a novel efficient cross-modality insulator augmentation algorithm for multi-domain insulator defect detection to mimic real complex scenarios. In this section, we will provide a detailed introduction to the proposed cross-modal insulator enhancement algorithm for multi-domain insulator defect detection. The multi-domain insulator defect detection framework is shown in Figure 1. The proposed cross-modal insulator enhancement algorithm is implemented by two modules: the high-resolution insulator cross-modal transformation (HICT) model and the multi-domain insulator multi-scale space enhancement (MMA) model. The HICT module aims to generate multi-modal insulator images with rich texture information to eliminate the adverse effects of existing modal differences. The MMA module is used to enhance multi-domain insulator images at different spatial scales simultaneously and utilizes these fused images and location information to help the target model more accurately locate defects of various scales. This algorithm improves the accuracy of multi-domain insulator defect detection without increasing inference resources, providing a new perspective for insulator defect detection in real-world scenarios.

### 3.1. The Definition of Multi-Domain Insulator Defect Detection

Insulators for transmission lines operating in outdoor environments are susceptible to failures caused by weather changes [2,26,27]. Using unmanned aerial vehicles to detect insulation defects can effectively and accurately assess the insulation condition. Most existing deep learning-based insulation defect detection models are trained using insulator images taken on sunny days, which may not be suitable for insulator detection tasks with diverse weather conditions. Compared to sunny days, insulators in foggy weather may suffer from poor lighting due to the blocking of light by fog, resulting in reduced image clarity. This may cause difficulties in using inspection models to detect insulators. During snowy weather, the surface of the insulator may condense water vapor or snowflakes, changing its shape and affecting the recognition ability of the detection model. In addition, heavy snow may cover the insulator, causing the model to fail to detect the target. Therefore, we cannot ignore the impact of season on the model. Many researchers have not incorporated this factor into insulator detection models, and existing datasets have limited reference value in this regard. To address these issues, we still need to use adversarial generative networks for style transfer to expand the types of data.

### 3.2. High-Resolution Insulator Cross-Modality Translation Module

Through using the insulator defect detection methods, it possible to quickly and accurately detect insulators and their defects in images, greatly saving manpower and material resources and improving maintenance efficiency. This is significant for ensuring the safe and effective operation of power grids. The early research on the formation mechanism of rain and fog was based on the method of establishing complex mathematical models to add noise to images. For example, references [20,22] used the dark channel prior to constructing foggy weather data. However, real weather conditions are very complex and diverse, and these traditional methods are not sufficient to simulate rich weather condition information, so it is difficult to achieve ideal results. Recently, the image translation approaches based on generative adversarial networks (GANs) [28,29] have achieved great success [30,31,32,33]. These methods aim to convert specific features of an image at the pixel level, achieving the transformation from the source domain to the target domain.

We propose a high-resolution insulator cross-modality translation (HICT) module designed to generate multi-modality insulator images with rich texture information to eliminate adverse effects of existing modality discrepancy, simulate real complex scenarios, and improve the robustness of insulation defect detection models under various weather conditions, thereby ensuring the accuracy and effectiveness of UAVs inspection.

**Figure 2:**
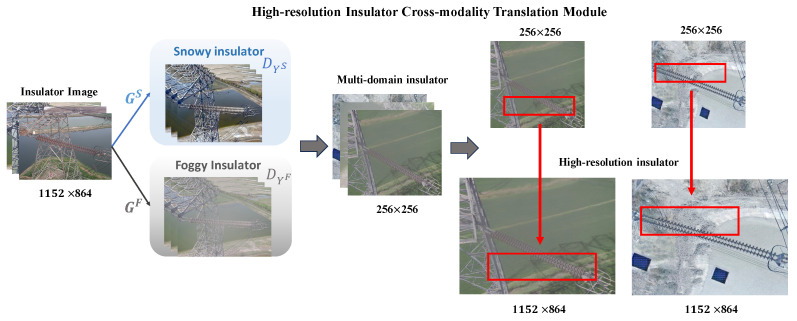
The high-resolution cross-modal translational model of insulators is divided into two parts. The original image of the insulator in the dataset is 1152×864 pixels, and a cross-modal translation model is used to output a 256×256 pixel multi-domain insulator image. Through high-resolution pixel reconstruction, an image of cross-modality translation insulators with the same size as the original image is obtained.

As shown in Figure 2, the weather conditions of the image range from clear skies to foggy conditions and then to snowy conditions, with the migration of the cross-modality translation insulator and subsequent pixel reconstruction resulting in high-resolution multi-domain insulator images. It is noted that the mentioned HICT module is a cascaded architecture (as shown in Figure 1), which mainly contains two models: a cross-modality generator and a high-resolution generator. We will give more details about them as follows.

In the first stage, the designed cross-modality generator aims to translate the raw insulator image into different weather-conditioned insulator images. The different weather-conditioned mapping function is denoted as $G^{N}:X\to Y^{N},N\in \left\{Snow,Fog\right\}$ and $F:Y^{N}\to X$. The source domain and the target domain are represented by *X* and *Y*, Snow and Fog mean snowy and foggy weather, respectively, and the two mappings are represented by *G* and *F*. Here, we choose the foggy weather as a representative for description convenience. Inspired by related work [29], we are given one set of images in domain *X* such as sunny weather insulators and a different set in domain *Y* such as foggy weather insulators. We may train a mapping G:X→Y such that the output $\hat{y}=G\left(x\right),x\in X$ is indistinguishable from images y∈Y by an adversary trained to classify $\hat{y}$ apart from *y*. The data *x* from the *X* domain are passed through the optimal generator *G* to obtain Fake $\hat{Y}$. Fake $\hat{Y}$ is passed through the inverse generator *F* to obtain the reconstructed result Fake $\hat{X}$. $D_{Y}$ and $D_{X}$ are associated adversarial discriminators. $D_{Y}$ encourages *G* to translate *X* into outputs indistinguishable from domain *Y* and vice versa for $D_{X}$ and *F*.

Our goal is to learn a mapping $G^{N}:X\to Y^{N},N\in \left\{Snow,Fog\right\}$ such that the distribution of images from $G\left(x\right)$ is indistinguishable from the distribution *Y* using an adversarial loss. Because this mapping is highly under-constrained, we couple it with an inverse mapping $F:Y^{N}\to X,N\in \left\{Snow,Fog\right\}$ and introduce a cycle consistency loss to enforce $F\left(G\left(x\right)\right)\approx x$ (and $G\left(F\left(y\right)\right)\approx y$). To further regularize the mappings, we introduce two cycle consistency losses that capture the intuition that if we translate from one domain to the other and back again, we should arrive at where we started: (1) forward cycle-consistency loss: $x\to G\left(x\right)\to F\left(G\left(x\right)\right)\approx x$; (2) backward cycle-consistency loss: $y\to F\left(y\right)\to G\left(F\left(y\right)\right)\approx y$. The objective contains two types of terms: adversarial losses [28] for matching the distribution of generated images to the data distribution in the target domain and cycle consistency losses [29] to prevent the learned mappings *G* and *F* from contradicting each other. The original adversarial loss formula is as follows:

(1) $$\begin{array}{cc} L_{GAN}\left(G,D_{Y},X,Y\right) & =E_{y∼p_{data\left(y\right)}}\left[logD_{Y}\left(y\right)\right]\\ \; & +E_{x∼p_{data(x)}}\left[log{(1-D}_{Y}\left(G(x)\right)\right], \end{array}$$

where *G* tries to generate images $G\left(x\right)$ that look similar to images from domain *Y*, while $D_{Y}$ aims to distinguish between translated samples $G\left(x\right)$ and real samples *y*. *G* aims to minimize this objective against adversary *D* that tries to maximize it. We introduce a similar adversarial loss for the mapping function F:Y→X and its discriminator $D_{X}$ as well.

For the image *x* from domain *X*, the image translation cycle should be able to bring *x* back to the original image, such as the forward cycle-consistency loss: $x\to G\left(x\right)\to F\left(G\left(x\right)\right)\approx x$. Similarly, for the image *y* from domain *Y*, the image translation cycle should be able to bring *y* back to the original image, such as the backward cycle-consistency loss: $y\to F\left(y\right)\to G\left(F\left(y\right)\right)\approx y$. For the mechanism to train stably, the cycle-consistency loss formula needs to be calculated as follows:

(2) $$\begin{array}{cc} L_{cyc}\left(G,F\right) & =E_{x∼p_{data\left(x\right)}}\;\left[{∥F\left(G\left(x\right)\right)-x∥}_{1}\right]\\ \; & +E_{y∼p_{data\left(y\right)}}\;\left[{∥G\left(F\left(y\right)\right)-y∥}_{1}\right]. \end{array}$$

To make the generated results consistent with the expected target category, we add identity loss to the discriminator. For the image *x* from domain *X*, we want the generated result to have both the visual effects of the target domain and the correct target category, that is $F\left(x\right)\approx x$. The identity loss is calculated for better performance as follows:

(3) $$\begin{array}{cc} L_{id}\left(G,F\right) & =E_{x∼p_{data\left(x\right)}}\;\left[{∥F\left(x\right)-x∥}_{1}\right]\\ \; & +E_{y∼p_{data\left(y\right)}}\;\left[{∥G\left(y\right)-y∥}_{1}\right]. \end{array}$$

As a consequence, the final loss formula of our model is presented as follows:

(4) $$\begin{array}{cc} L_{total}\left(G,D_{Y},F,D_{X},\right) & =E\left[logD_{Y}\left(y\right)\right]+E\left[log{(1-D}_{Y}\left(G(x)\right)\right]+E\left[logD_{X}\left(x\right)\right]\\ \; & +E\left[log{(1-D}_{X}\left(G(Y)\right)\right]+\lambda L_{cyc}+L_{id}. \end{array}$$

Here, λ controls the relative importance of *G* and *F*, which means that the generator *G* should achieve the transfer from *X* to *Y* as much as possible, and the generator *F* should achieve the transfer from *Y* to *X* as much as possible. At the same time, it is hoped that the two generators can achieve reciprocity: that is, they can iteratively return to themselves. We only select *G* as the cross-modality insulator generator model, which can translate the raw images into different weather-conditioned insulator images.

In the second training stage, we aim to leverage the high-resolution insulator generator to enrich the texture and location information for better detection performance. Based on the network architecture of cross-modality translation, the output image size is only 256×256 pixels. Motivated by the work [34], we train the super-resolution model with extra public datasets (DIV2K dataset and Flickr2K dataset). With the same training protocols, we utilize the mentioned degradation-aware block (as shown in Figure 1) to fuse learned degradation representation and the ${∥\cdot ∥}_{1}$ loss to constrain high-quality generated images. The pre-trained super-resolution model is chosen as the high-resolution insulator generator Φ. Finally, we can generate high-quality cross-modality insulator images through the inference Φ(G(x)).

### 3.3. Multi-Domain Insulator Multi-Scale Spatial Augmentation Module

**Figure 3:**
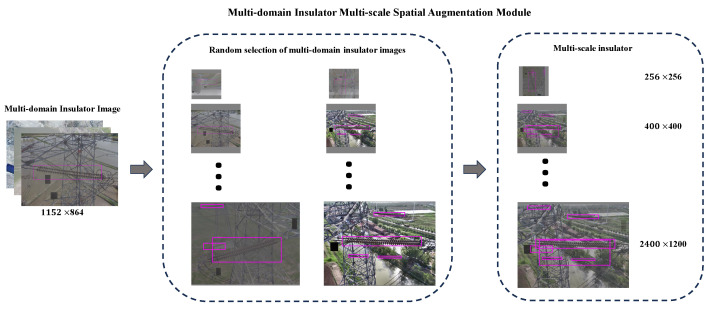
Schematic illustration of multi-domain insulator multi-scale spatial augmentation module. Randomly select two images from the multi-domain insulator datasets, fusing both images and bounding boxes in different spatial scales.

After the former mentioned HICT module, we can convert a sunny original insulator image *x* into a high-quality multi-domain insulator image Φ(G(x)). In other words, the original domain *X* can be translated into the hybrid domain *H*, which contains sunny, snowy, and foggy weather domains. In this section, we will perform multi-domain insulator multi-scale spatial augmentation based on the multi-domain insulator data transformed by the HICT module, which can help improve the model’s recognition ability in small target detection. In the field of object recognition, data augmentation can be constructed by flipping, rotating, and scaling the original samples. Different from them, the multi-domain insulator multi-scale spatial augmentation module is proposed as shown in Figure 3. It reduces the model’s reliance on noisy samples to minimize the impact of noisy samples on the model.

The fusion formula for augmentation samples is as follows:

(5) $$\begin{array}{c} \tilde{h}=\alpha h_{i}+\left(1-\alpha \right)h_{j},\;where\;h\in H,i\neq j.\\ \tilde{b}=\left[b_{i},b_{j}\right],where\;i\neq j. \end{array}$$

Here, *b* means the bounding boxes for insulator images. The parameter α is set as 0.5 as default. The bicubic interpolation operation is utilized to generate different-scale augmented data. We aim to fuse different scale insulator images (as shown in Figure 3) in the hybrid domain to improve the generalization ability in scale variance and modality variance scenarios. In the MMA module, we select 30% of the insulator images for data augmentation by scaling and mix-up. We choose the different scale at 256×256, 400×400, 800×800, 600×800, 1200×800, and 2400×1200 to construct a new dataset for multi-domain insulator defect detection.

In general, we propose the high-resolution insulator cross-modality translation (HICT) module designed to generate multi-modality insulator images with rich texture information to eliminate adverse effects of existing modality discrepancy. The multi-domain insulator multi-scale spatial augmentation (MMA) module simultaneously augments multi-domain insulator images with different spatial scales and leverages these fused images and location information to help the target model locate defects with various scales more accurately. The method addresses the lack of research on data augmentation strategies in the task of insulator defect detection. It also alleviates the overfitting problem without increasing the inference resources. Finally, we can choose a suitable lightweight object detection model as follows. This efficient cross-modality insulator augmentation algorithm can be used for multi-domain insulator defect detection and is more suitable for real complex scenarios.

### 3.4. The System of Evaluation Indicator in Insulator Defect Detection Model

In target detection algorithms [22,35,36], we use commonly used evaluation metrics such as average precision (AP) and mean average precision (mAP) as evaluation metrics in this article. The calculation formula is as follows:

(6) $$\begin{array}{cc} AP & =\frac{1}{m}\sum_{i}^{m}P_{i}\\ \; & =\frac{1}{m\;}*P_{1}+\frac{1}{m\;}*P_{2}+\ldots +\frac{1}{m\;}*P_{m}\\ \; & =\int P\left(R\right)dR\;, \end{array}$$

where *R* is recall, and *P* is precision; AP is the average precision for a certain class of n samples, assuming it has m positive examples, each positive example corresponds to a recall *R* value $\left(\frac{1}{m},\frac{2}{m},\cdots ,1\right)$, and the maximum precision *P* is calculated for each recall. Then, the mean of these *P* values is taken. The mean of all AP for each class in the dataset is taken to obtain mAP:

(7) $$mAP=\frac{1}{C}\sum_{j}^{C}{AP}_{j}$$

where *P* is precision, AP is the average precision of a class of samples, and mAP is the average precision of the dataset. The evaluation indicators used in this article are mAP@50:95, mAP@50, and mAP@75. mAP@50:95 refers to the mAP calculated with Intersection over Union (IoU) values ranging from 50% to 95% with a step size of 5%. mAP@50, and mAP@75 represent the mAP values with IoU of 0.5 and 0.75, respectively.

## 4. Experiments

In this section, we evaluated the proposed multi-domain insulator defect detection on several public insulator databases: the Unifying Public Insulator Datasets (UPID) dataset [37], the Synthetic Foggy Insulator Dataset (SFID) dataset [20]. We compared other popular methods, and the experimental results prove that our method achieved satisfactory performance in the multi-domain insulator defect detection task. Then, we investigate the effect of different parameters on the recognition performance. Finally, we conduct the ablation study to evaluate the effectiveness of the proposed HICT and MMA modules.

### 4.1. Databases

The CPLID dataset [38] is provided by the State Grid Corporation of China and obtained from real transmission line scenarios using drone inspection. It contains 600 images of normal insulators and 248 images of insulator defects. The UPID dataset is obtained by Andrea et al. [37] through methods such as random affine transformation, Gaussian blurring, and lossless transformation. It consists of 6860 training and testing images; we randomly selected 80% of the insulator images as the training set, and the remaining ones were used as the testing set. The SFID dataset [20] contains 13,718 images including synthetic foggy images and uses random brightness and fog thickness to enhance the UPID dataset. We randomly selected 80% of the insulator images as the training set, and the remaining ones were used as the testing set.

**Figure 4:**
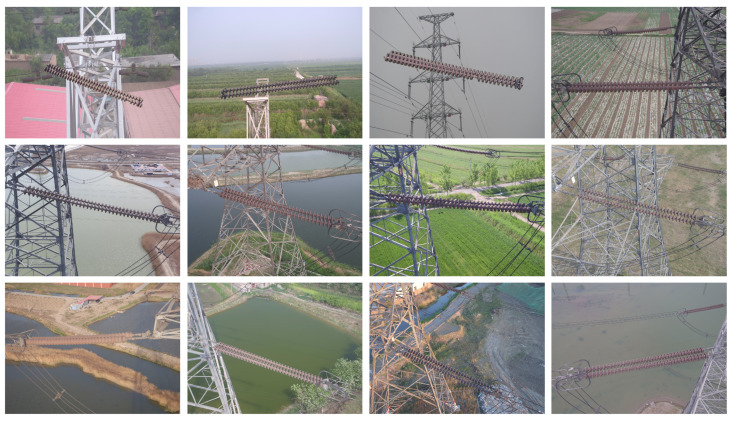
The illustration of public representative transmission line insulator databases.

In the following experiments, the original data we used were insulator images obtained from drone inspections in real scenes. Some of them were from the CPLID dataset and SFID dataset, and some were provided by relevant power workers. There were a total of 10,974 original images. Example insulator images are shown in Figure 4. Then, we used the HITC model and MMA module for cross-modality data augmentation to generate multi-modality insulator images. During model training, we found that as the amount of generated data increases, the accuracy of the detection model also increases. To avoid overfitting, we used twice as much generated data as the original data. The final obtained images of 21,097, the training, validation, and test sets for the network model were trained according to a ratio of 7:2:1.

### 4.2. Implementation Details

This paper carries out the experiments in this study on a platform using an Ubuntu18.04 system, Nvidia RTX 3060 GPU with a memory of 24G. We utilize the Faster R-CNN model [39] as the backbone network in the multi-domain insulator defect detection module. The method is implemented based on the PyTorch deep learning platform. The multi-domain insulator defect defection model is trained on a limited 100 epochs with a batch size of 16 and a learning rate of 0.001.

In the HITC model for cross-modal data augmentation to generate multi-modality insulator images with rich texture information, the dataset contained insulator images in sunny weather conditions, insulator images in foggy weather conditions, and insulator images in snowy weather conditions, eliminating the adverse effects of existing modal differences. All images were adjusted to 256×256, and all networks were trained from scratch using the Adam solver [29,40] with a batch size of 1 and a learning rate of 0.0002. We keep the same learning rate for the first 100 epochs and linearly decay the rate to zero over the next 100 epochs. In the cyclic consistency loss (Section 3.2), we set λ=10 in Formula (4). Specifically, in the HICT module, we use the SFID dataset to train the fog weather transformation model for insulator migration and the summer2winter dataset for insulator-style transfer in snowy weather. In the MMA module, we select 30% of the insulator images for data enhancement at 256×256, 400×400, 800×800, 600×800, 1200×800, and 2400×1200 scales, and then we input all the images into the training model for further evaluation to simultaneously augment multi-domain insulator images with different spatial scales and leverage these fused images and location information to help the target model locate defects with various scales more accurately.

### 4.3. Comparison Experiment

The insulator and defect detection results of different frameworks are shown in Table 1.

To verify the effectiveness of the proposed method in this article, we conducted comparative experiments using different detection models under the same conditions on the detectron2 framework. Table 1 demonstrates the comparative experiments of this method with popular object detection algorithms in the past five years in terms of multi-domain insulator target detection capabilities. In the experiments, we found that when using the SFID dataset containing insulator data from sunny and foggy weather conditions for training, the YOLOv5 detection model achieved the best detection accuracy of 96% in terms of mAP@50 for multi-domain insulators. YOLOv8 and Faster R-CNN detection models had similar mAP@50 accuracy rates of over 91%. The Faster R-CNN model had a higher detection accuracy in terms of mAP@50:95 with a rate of 77.43%. Although the YOLOv5 detection model had the best detection accuracy in terms of mAP@50, the model had lower accuracy rates in both mAP@50 and mAP@50:95 compared to other models. Therefore, we selected Faster R-CNN as the main network for this experiment. When we use the same Faster R-CNN backbone as the detection model for testing, the results show that the cross-modality insulator augmentation (CMIA) algorithm proposed in this paper can effectively improve the ability of the detection model to detect insulators in a multi-domain environment, the mAP@50 metric increased from 91.98% to 98.48%, surpassing the accuracy of YOLOv5 detection model and reaching the best accuracy. The mAP@75 metric increased from 86.96% to 94.45%, and the mAP@50:95 metric increased from 77.43% to 96.17%. The results show that using the cross-modality insulator augmentation algorithm proposed in this paper, the multi-domain insulator defect detection model can achieve a prediction accuracy of over 98%, effectively improving the model’s recognition rate of insulators under extreme weather conditions, making it more valuable in the real world.

### 4.4. Ablation Study

The proposed multi-domain insulator defect detection framework mainly contains two designed modules: the high-resolution insulator cross-modality translation (HICT) module and the multi-domain insulator multi-scale spatial augmentation (MMA) module. To reveal how each module contributes to performance improvement, we conduct a comprehensive ablation study to analyze them on the UPID insulator dataset, as shown in Table 2.

The performance of the proposed method variants is summarized in Table 2 on the UPID dataset containing only sunny weather condition insulator data. We utilize the pure Faster RCNN algorithm as the baseline method for a fair comparison. Because of the limited weather mode gap in the insulator dataset, the baseline performance of the insulator defect detection model task is poor. However, through the designed HICT model, the mAP@50 metric increased from 90.52% to 98.34%, and the mAP@75 metric significantly increased from 87.79% to 94.41%. This is because our designed HICT model generates multi-modality insulator images with rich texture information to eliminate adverse effects of existing modality discrepancy. When the additional MMA strategy is used, the Insulator mAP@50:95 accuracy increases from 80.32% to 88.39%, and the Defect mAP@50:95 accuracy increases from 74.52% to 84.27%, achieving the best recognition performance for insulator defects. This benefits from the simple MMA algorithm to simultaneously augment multi-domain insulator images with different spatial scales and leverage these fused images and location information to help the target model locate defects with various scales more accurately.

Through ablation study analysis, these important theoretical findings are proved as follows: 1. We found that using the HITC model can effectively improve the detection accuracy of the detection model for multi-domain insulators. As the amount of generated data increases, the accuracy of the detection model also increases. 2. We found that using the MMA model can further improve the detection accuracy of the model, especially for the detection of small target defects in the manuscript. When the amount of generated data is increased to double the original data, the accuracy still increases. 3. We believe that appropriately increasing the amount of generated data can effectively alleviate overfitting and improve the detection accuracy of the model.

### 4.5. Cross-Dataset Evaluation

The analysis of the ablation study in the previous section shows that high-quality multi-domain and multi-scale insulator models not only make the detection model more robust but also improve overall performance. In this section, we test the performance of the proposed method on the SFID and UPID test sets. SFID is a single-scale test set with foggy conditions, including 4318 insulators and 760 self-explosion defects. UPID is a single-scale test set containing only data on insulators in clear weather, including 4318 insulators and 760 self-explosion defects. These models are trained in a limited 100 periods.

The performance of our proposed method on different datasets is shown in Table 3. The results show that the performance of our proposed method is better in the more complex weather scenarios, and its performance on the test set SFID with foggy weather is better than that of the UPID test set with only sunny data. On the test set SFID, it achieves mAP@50:95, mAP@50, and mAP@75 scores of 98.48%, 96.17%, and 86.33%, respectively, which are higher than the UPID metrics for the test set. The experimental results show that the proposed multi-domain insulator defect detection algorithm can also achieve satisfactory recognition performance on other test sets.

### 4.6. Algorithm Analysis

**Figure 5:**
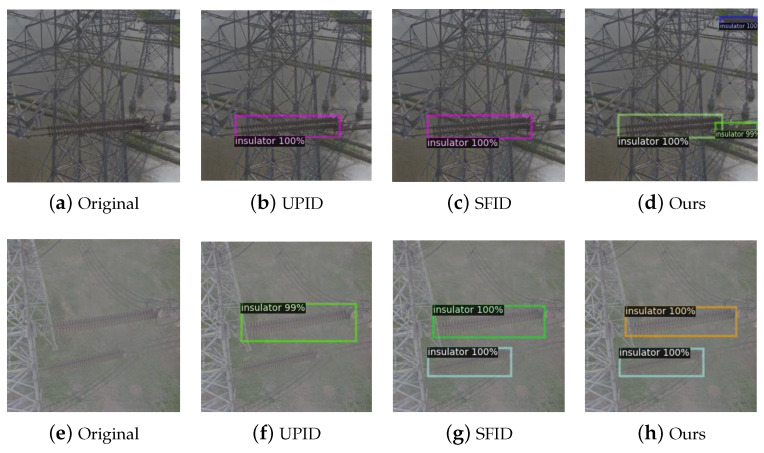

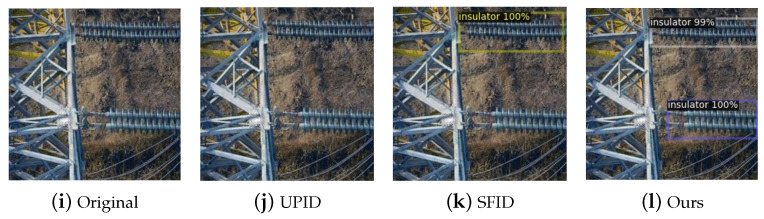
Detection results of insulator detection models on different datasets. The first row shows the insulator detection results under overcast conditions using the UPID, SFID, and our training sets, respectively. The second row displays the insulator detection results under foggy conditions using the UPID, SFID, and our training sets, respectively. The last row presents the insulator defect detection results under snowy conditions using the UPID, SFID, and our training sets, respectively.

We tested a set of insulator detection models under different weather conditions, as shown in Figure 5. The first column on the left shows the original test images, which simulate three different weather scenarios, including cloudy, foggy, and snowy. The second column on the left shows the detection results recorded by the model during UPID training. The second column on the right is the detection results recorded by the model during SFID training; the right column 1 is the test results of the PTID training method proposed in this article. The results in Figure 5 show that the model trained using the SFID dataset is superior to the model trained using the UPID dataset for insulator detection in foggy conditions. However, the model trained using the SFID dataset cannot detect overlapping insulators and distant insulator strings in close-up views under cloudy conditions, and its detection capability is significantly lower than that of the model trained using the dataset proposed in this paper. The model trained using the dataset proposed in this paper produces the best detection results, especially in cloudy and snowy scenes with stronger advantages.

Through the above experimental analysis, we found that using the high-resolution insulator cross-modality translation (HICT) module can effectively improve the detection accuracy of the detection model for multi-domain insulators, and using the multi-domain insulator multi-scale spatial augmentation (MMA) module can effectively alleviate the problem of missed insulator detections in multi-scale scenarios. As the amount of generated data increases, the accuracy of the detection model also increases. When the generated data continue to increase to double the original data, the accuracy still increases. Appropriately increasing the amount of generated data can effectively alleviate overfitting and improve the detection accuracy of the model.

## 5. Conclusions

Transmission line insulators operate outdoors and are prone to damage from various weather conditions. Therefore, the designed insulator defect detection model requires good generalization ability to adapt multi-domain insulator processing. The paper proposes a novel efficient cross-modality insulator augmentation algorithm for multi-domain insulator defect detection to mimic real complex scenarios. The proposed high-resolution insulator cross-modality translation module can effectively generate high-quality insulator images with various weather conditions (e.g., foggy and snowy), which can boost detection precision in a complex and unstable environment. Additionally, we design the multi-domain insulator multi-scale spatial augmentation algorithm to fuse images and bounding boxes with different scales to enhance the detection ability for various-scale insulator defects. It has high detection accuracy on multiple public insulator datasets (UPID and SFID insulator defect datasets), especially on the SFID dataset, where the insulator defect detection accuracy is as high as 98.48%, which is 6.78% higher than that of the insulator defect detection algorithm based on Yolov8. This experimental result demonstrates the superior insulator defect detection performance of our method. In the future, we will evaluate the proposed method’s performance on more complex multi-domain insulator datasets and explore better robustness to mimic real-world scenarios.

## Figures and Tables

**Table 1:** A comparison of the insulator defect detection algorithms.

Train Data	Model	mAP@50 (%)	mAP@75 (%)	mAP@50:95 (%)
SFID [20]	Mask RCNN [41]	91.39	86.21	75.19
	Rentinanet [42]	92.57	84.93	73.62
	Fast RCNN [43]	92.15	84.53	72.27
	Faster RCNN [39]	91.98	86.96	77.43
	YOLOv5 [44]	96	81.1	69.1
	YOLOv8 [45]	91.7	87.1	76.7
CMIA	Ours	98.48 *	94.45 *	96.17 *

* means the best detection result in Table 1.

**Table 2:** The ablation study on the UPID dataset where the baseline utilizes a pure Faster RCNN model.

Baseline	HICT	MMA	mAP@50 (%)	mAP@75 (%)	mAP@50:95 (%)	mAP@50:95 Insulator (%)	mAP@50:95 Defect (%)
✔	-	-	90.52	87.79	77.42	80.32	74.52
✔	✔	-	98.34	94.41	81.51	82.95	80.04
✔	✔	✔	98.48 *	96.17 *	86.33 *	88.39 *	84.27 *

✔ means the module utilized in the experiment; * means the best detection result in Table 2.

**Table 3:** Cross-database testing accuracies (%) of the proposed approach using UPID and SFID.

Testing	Classes	Number	mAP@50 (%)	mAP@75 (%)	mAP@50:95 (%)
UPID	insulator	2076	-	-	84.91
	defect	371	-	-	80.72
	average	2447	97.38	94.95	82.82
SFID	insulator	4318	-	-	88.39
	defect	760	-	-	84.27
	average	5078	98.48	96.17	86.33

## Data Availability

The Unifying Public Datasets for Insulator (UPID) can be downloaded from https://github.com/heitorcfelix/public-insulator-datasets (accessed on 13 June 2022); The synthetic foggy insulator dataset (SFID) can be downloaded from https://github.com/zhangzhengde0225/FINet; And the Summer2Winter Yosemite dataset can be downloaded from https://www.kaggle.com/datasets/balraj98/summer2winter-yosemite.

## Abbreviations

The following abbreviations are used in this manuscript:

UAV	Unmanned Aerial Vehicle
SCNN	Siamese Convolutional Neural Network
SSD	Single-Shot MultiBox Detector
Faster R-CNN	Faster Region-Based Convolutional Neural Network
YOLO	You Only Look Once
CNN	Convolutional Neural Network
UPID	Unifying Public Insulator Datasets
SFID	Synthetic Foggy Insulator Dataset
CPILD	Chinese Power Line Insulator Dataset
MMA	Multi-Domain Insulator Multi-Scale Spatial Augmentation
HICT	High-Resolution Insulator Cross-Modality Translation
CMIA	Cross-Modality Insulator Augmentation
